# Supplementary material for: Gene expression studies of WT1 mutant Wilms tumor cell lines in the frame work of published kidney development data reveals their early kidney stem cell origin
Source: PLoS One. 2023 Jan 23;18(1):e0270380. doi: 10.1371/journal.pone.0270380 (PMC9870146; doi:10.1371/journal.pone.0270380)
Supplement: S1 File — (PDF) [file pone.0270380.s001.pdf]

Supplementary Figures S1-S9

A

1: GO: Molecular Function [Display Chart] 203 input genes in category / 595 annotations before applied cutoff / 19415 genes in category

	ID	Name	Source	pValue	FDR B&H	FDR B&Y	Bonferroni	Genes from Input	Genes in Annotation
1	GO:0050839	cell adhesion molecule binding		3.460E-5	2.059E-2	1.434E-1	2.059E-2	18	573
2	GO:0005102	signaling receptor binding		8.021E-5	2.386E-2	1.662E-1	4.772E-2	37	1842
3	GO:0044877	protein-containing complex binding		1.900E-4	3.438E-2	2.395E-1	1.130E-1	32	1565
4	GO:0017154	semaphorin receptor activity		2.311E-4	3.438E-2	2.395E-1	1.375E-1	3	12
5	GO:0003779	actin binding		3.300E-4	3.927E-2	2.736E-1	1.964E-1	14	456

Show 1 more annotation

Highly expressed genes in Wilms cell lines*

BCAM, PFKP, PTRF, EZR, KRT18, DSP
CHN1, S100A4, SLIT2, TUBB3, UCHL1, EDARADD, PDGFC

2: GO: Biological Process [Display Chart] 200 input genes in category / 4443 annotations before applied cutoff / 20213 genes in category

	ID	Name	Source	pValue	FDR B&H	FDR B&Y	Bonferroni	Genes from Input	Genes in Annotation
1	GO:0000902	cell morphogenesis		3.539E-11	1.572E-7	1.411E-6	1.572E-7	39	1197
2	GO:0030029	actin filament-based process		7.384E-10	1.640E-6	1.472E-5	3.281E-6	31	879
3	GO:0051270	regulation of cellular component movement		1.759E-9	1.674E-6	1.502E-5	7.817E-6	37	1250
4	GO:0016477	cell migration		1.838E-9	1.674E-6	1.502E-5	8.165E-6	46	1812
5	GO:0032989	cellular component morphogenesis		1.884E-9	1.674E-6	1.502E-5	8.369E-6	31	914

Show 45 more annotations

B

1: GO: Molecular Function [Display Chart] 492 input genes in category / 957 annotations before applied cutoff / 19415 genes in category

	ID	Name	Source	pValue	FDR B&H	FDR B&Y	Bonferroni	Genes from Input	Genes in Annotation
1	GO:0003779	actin binding		7.050E-7	6.747E-4	5.020E-3	6.747E-4	31	456
2	GO:0050839	cell adhesion molecule binding		1.113E-5	5.325E-3	3.963E-2	1.065E-2	33	573
3	GO:0005102	signaling receptor binding		2.543E-5	8.113E-3	6.037E-2	2.434E-2	75	1842
4	GO:0098631	cell adhesion mediator activity		4.645E-5	1.111E-2	8.269E-2	4.445E-2	9	67
5	GO:0098632	cell-cell adhesion mediator activity		8.921E-5	1.707E-2	1.271E-1	8.537E-2	8	57

Show 4 more annotations

Highly expressed genes huNPC#

BCAM, PFKP, EZR, PTPRF, KRT18
UCHL1, PDGFA, CHN1, REEP2, S100A4, TUBB3,

2: GO: Biological Process [Display Chart] 484 input genes in category / 6255 annotations before applied cutoff / 20213 genes in category

	ID	Name	Source	pValue	FDR B&H	FDR B&Y	Bonferroni	Genes from Input	Genes in Annotation
1	GO:0000902	cell morphogenesis		1.493E-16	7.251E-13	6.757E-12	9.337E-13	79	1197
2	GO:0034330	cell junction organization		2.319E-16	7.251E-13	6.757E-12	1.450E-12	63	820
3	GO:0022008	neurogenesis		7.378E-16	1.538E-12	1.434E-11	4.615E-12	105	1940
4	GO:0000904	cell morphogenesis involved in differentiation		2.960E-15	4.629E-12	4.313E-11	1.851E-11	64	889
5	GO:0032989	cellular component morphogenesis		3.701E-14	4.630E-11	4.314E-10	2.315E-10	63	914

Show 45 more annotations

S1 Fig. Analysis of genes from the enriched NPC compartment expressed in common in Wilms cells [32]. A) ToppGene analyses of the 207 NPC enriched genes expressed in common in Wilms cells. *: genes, with an expression level between 3000 and 260 000 **C)** ToppGene analysis of all 534 normal huNPC enriched genes [32]. #: genes with a mean TPM (transcript per million), between 10 and 172 [32].

A

Highly expressed genes in Wilms cell lines*

1: GO: Molecular Function [Display Chart] 348 input genes in category / 746 annotations before applied cutoff / 19415 genes in category

	ID	Name	Source	pValue	FDR B&H	FDR B&Y	Bonferroni	Genes from Input	Genes in Annotation
1	GO:0005201	extracellular matrix structural constituent		1.180E-24	8.801E-22	6.330E-21	8.801E-22	34	185
2	GO:0005198	structural molecule activity		1.169E-14	4.360E-12	3.136E-11	8.721E-12	48	743
3	GO:0005178	integrin binding		7.906E-13	1.966E-10	1.414E-9	5.898E-10	21	157
4	GO:0030020	extracellular matrix structural constituent conferring tensile strength		6.214E-12	1.159E-9	8.336E-9	4.636E-9	12	42
5	GO:0050839	cell adhesion molecule binding		6.921E-11	1.033E-8	7.427E-8	5.163E-8	36	573

Show 45 more annotations

COL1A2, COL4A2, FN1, IGFBP7, DCN, TGFBI

B

Highly expressed genes huIPC#

1: GO: Molecular Function [Display Chart] 471 input genes in category / 902 annotations before applied cutoff / 19415 genes in category

	ID	Name	Source	pValue	FDR B&H	FDR B&Y	Bonferroni	Genes from Input	Genes in Annotation
1	GO:0005201	extracellular matrix structural constituent		1.454E-25	1.312E-22	9.685E-22	1.312E-22	39	185
2	GO:0030020	extracellular matrix structural constituent conferring tensile strength		2.575E-14	1.161E-11	8.574E-11	2.323E-11	15	42
3	GO:0005198	structural molecule activity		1.655E-13	4.976E-11	3.673E-10	1.493E-10	55	743
4	GO:0005178	integrin binding		4.728E-12	1.066E-9	7.871E-9	4.265E-9	23	157
5	GO:0050839	cell adhesion molecule binding		6.830E-10	1.232E-7	9.096E-7	6.160E-7	41	573

Show 45 more annotations

COL1A2, COL4A2, POSTN, EMILIN1, TGFBI , IGFBP7

S2 Fig. Functional analysis of genes of the IPC enriched genes [32]. A) ToppGene analyses of the 360 IPC enriched genes also expressed in Wilms cells >1000. *: genes with an expressed level between 20 000-270 000 in Wilms cell lines. **B)** ToppGene analysis of all 503 normal huIPC enriched genes [32]. #: genes with a mean TPM between 70 and 281 [32].

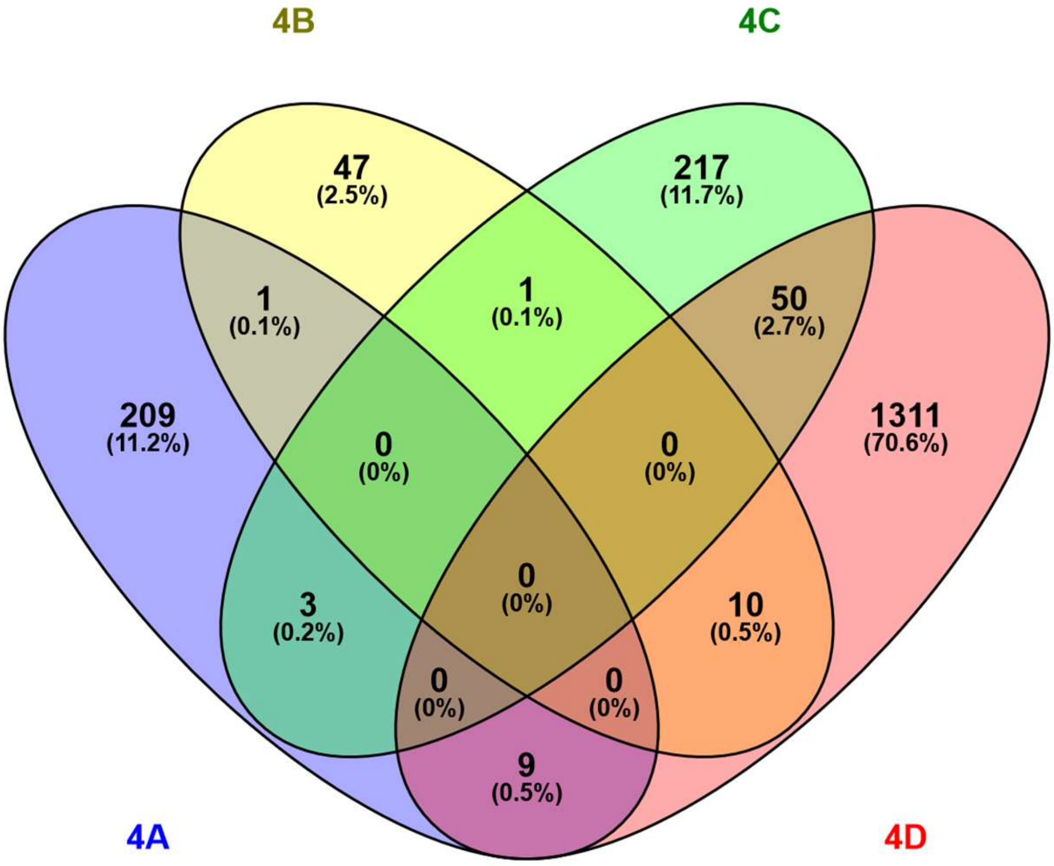

S3 Fig. Overlapping genes from the normal human nephrogenic clusters 4A, 4B, 4C and 4D [32].

Cluster 4A ,corresponds to 222 genes, 209 are exclusively expressed; cluster 4B, corresponds to 59 genes and 47 are exclusive; cluster 4C corresponds to 271 genes and 217 are exclusive; cluster 4D corresponds to 1380 genes and 1311 are exclusive

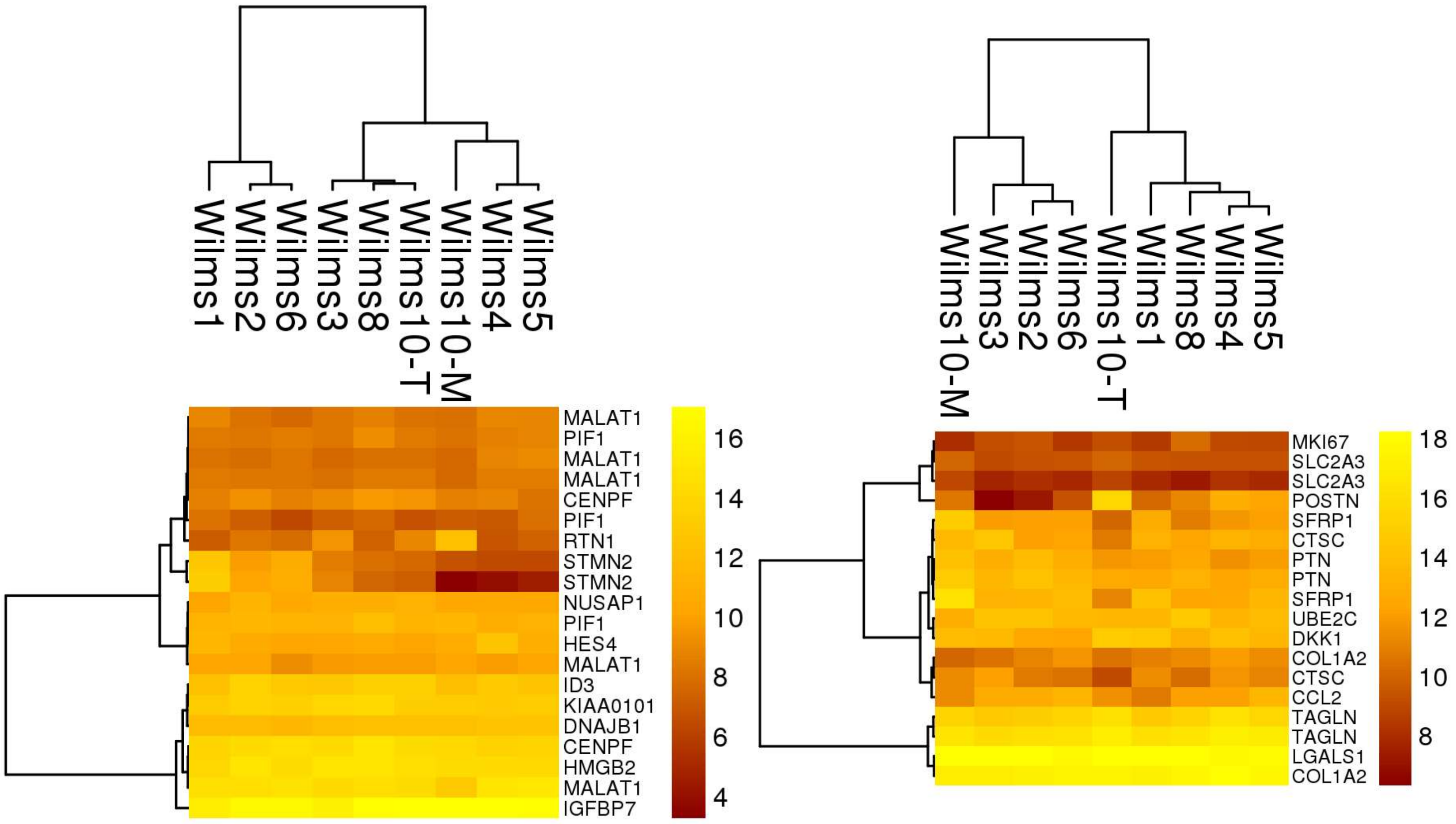

ID3	NPCa	
TMEM100	NPCa & NPCc	
KIAA0101	NPCa & IPC	
HSPA6	NPCa	2/5
MALAT1	NPCa	
RP11-609N14.1	NPCa	
DNAJB1	NPCb & NPCc	
CA7	NPCb	1/2
CTD-3126B10.4	NPCb	
RTN1	NPCc	
PTH	NPCc	3/4
IGFBP7	NPCc	
HES4	NPCc	
STMN2	NPCd	
CENPF	NPCd	
NUSAP1	NPCd	5/5
PIF1	NPCd	
HMGB2	NPCd	

LGALS1	IPC & ICa	
PTN	IPC	4/4
MKI67	IPC	
UBE2C	IPC	
SLC2A3	ICa	
DKK1	ICa	
CCL2	ICa	6/6
POSTN	ICa	
TAGLN	ICa	
COL1A2	ICa	
SFRP1	ICb	
SULT1E1	ICb	2/3
CTSC	ICb	

S4 Fig. *KeyGenes* expressed in Wilms cells corresponding to the different kidney clusters [38]. Heat maps of the expressed *KeyGenes* in Wilms cell with lists of all marker genes from each normal human cluster below the maps [38]. If a gene is also expressed in another cluster this is listed in the same line. Expression of genes in Wilms cell lines of all clusters are shown as heat maps. Symbols from BACs e.g., RP11-609N14.1 were not included in the calculations as no genes were represented by these.

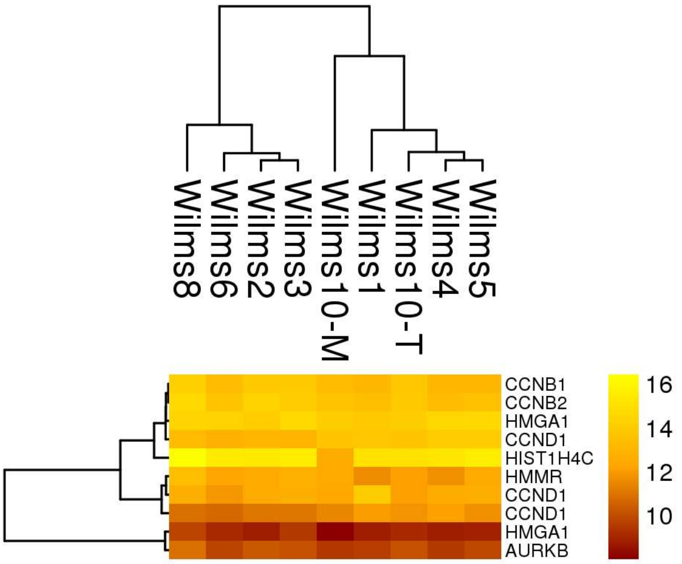

HMMR	PTA		
CCND1	PTA & RVCSBa & RVCSBb		
CALCA	PTA & RVCSBa		
CCNB1	PTA		7/8
HMGA1	PTA		
CCNB2	PTA		
AURKB	PTA		
HIST1H4C	PTA		

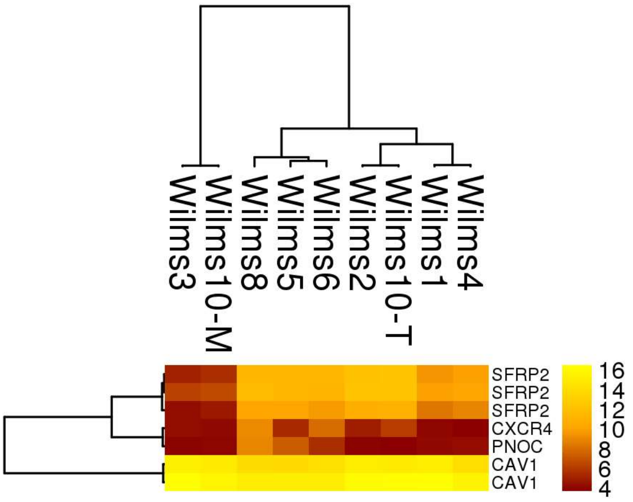

CRYM	RVCSBa		
CXCR4	RVCSBa		2/3
SFRP2	RVCSBa & RVCSBb		
CAV1	RVCSBb		
OLFM3	RVCSBb & SSBpod		
ERP27	RVCSBb		2/5
KAAG1	RVCSBb		
PNOC	RVCSBb		

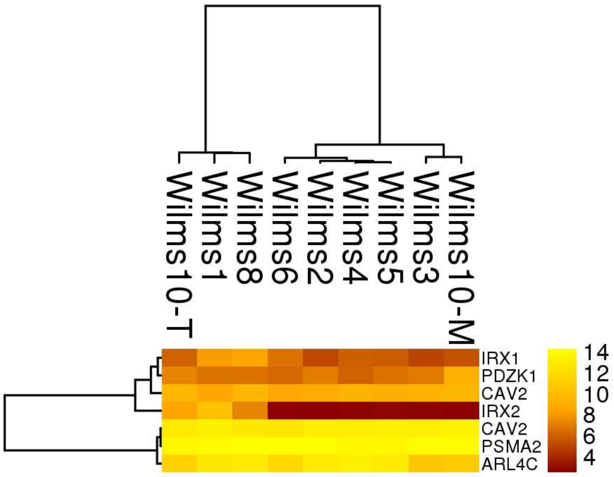

TSPAN15	SSBm/d	
PAPPA2	SSBm/d	
IRX1	SSBm/d	2/5
IRX2	SSBm/d	
WNT7B	SSBm/d	
LGALS2	SSBpr	
CAV2	SSBpr	
PSMA2	SSBpr	4/5
PDZK1	SSBpr	
ARL4C	SSBpr	
RP11-295M3.4	SSBpr	

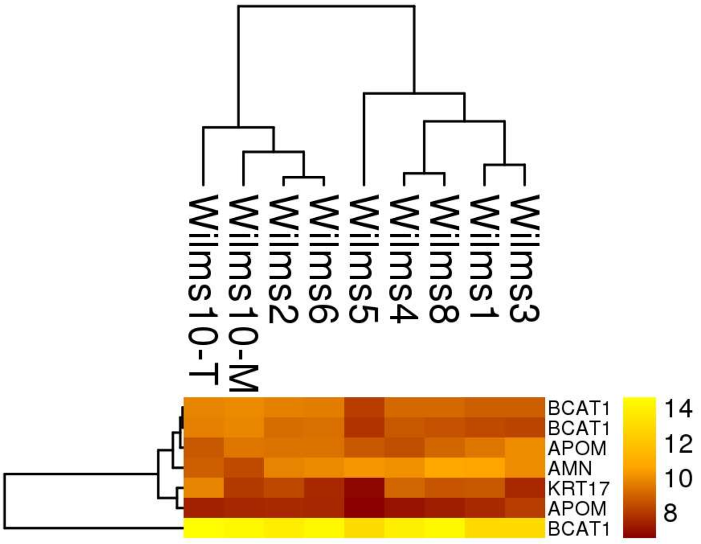

BCAT1	CnT	2/2
KRT17	CnT	
SLC12A1	DTLH	0/4
ATP6V1B1	DTLH	
KCNJ1	DTLH	
DEFB1	DTLH	
CUBN	ErPrT	2/6
HAO2	ErPrT	
APOA1	ErPrT	
GLYAT	ErPrT	
AMN	ErPrT	
APOM	ErPrT	

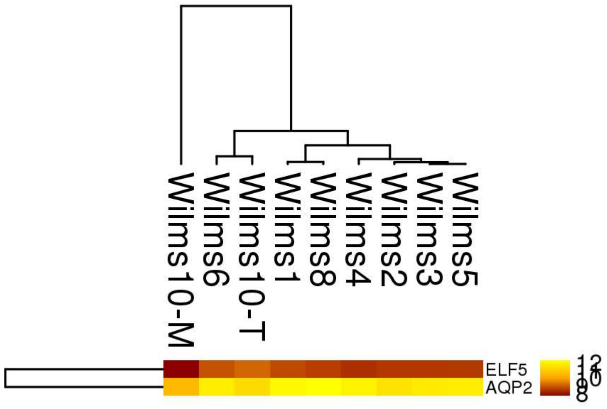

AOC1	UBCD	2/5
AGR2	UBCD	
ELF5	UBCD	
AQP2	UBCD	
SMIM22	UBCD	

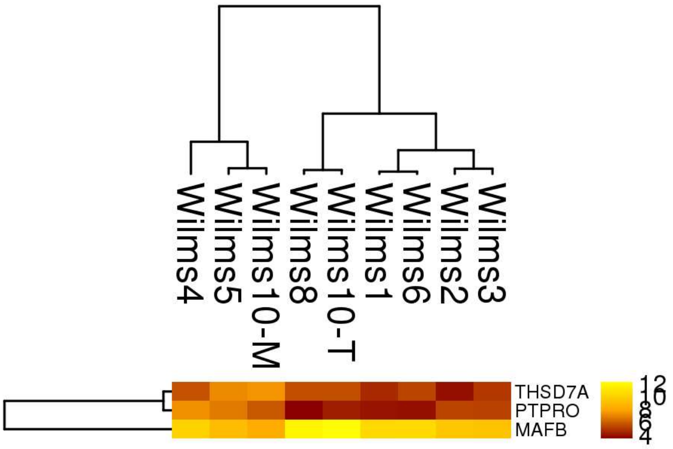

THSD7A	Pod	3/4
NPHS2	Pod	
PTPRO	Pod	
MAFB	Pod	

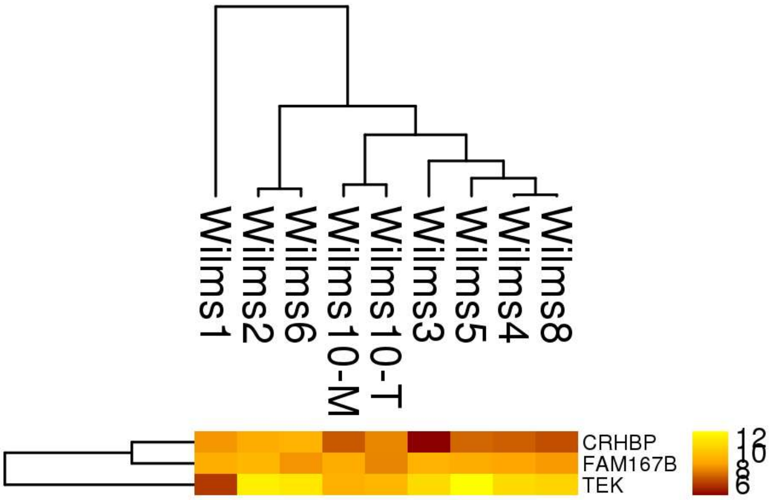

HIGD1B	Mes	0/2
REN	Mes	
TEK	End	3/8
APLNR	End	
RBP5	End	
CRHBP	End	
TM4SF18	End	
GIMAP7	End	
FAM167B	End	
KANK3	End	
LYZ	Leu	0/4
NCF4	Leu	
CD48	Leu	
LST1	Leu	
MT-CYB	Prolif	unknown
MT-CO1	Prolif	

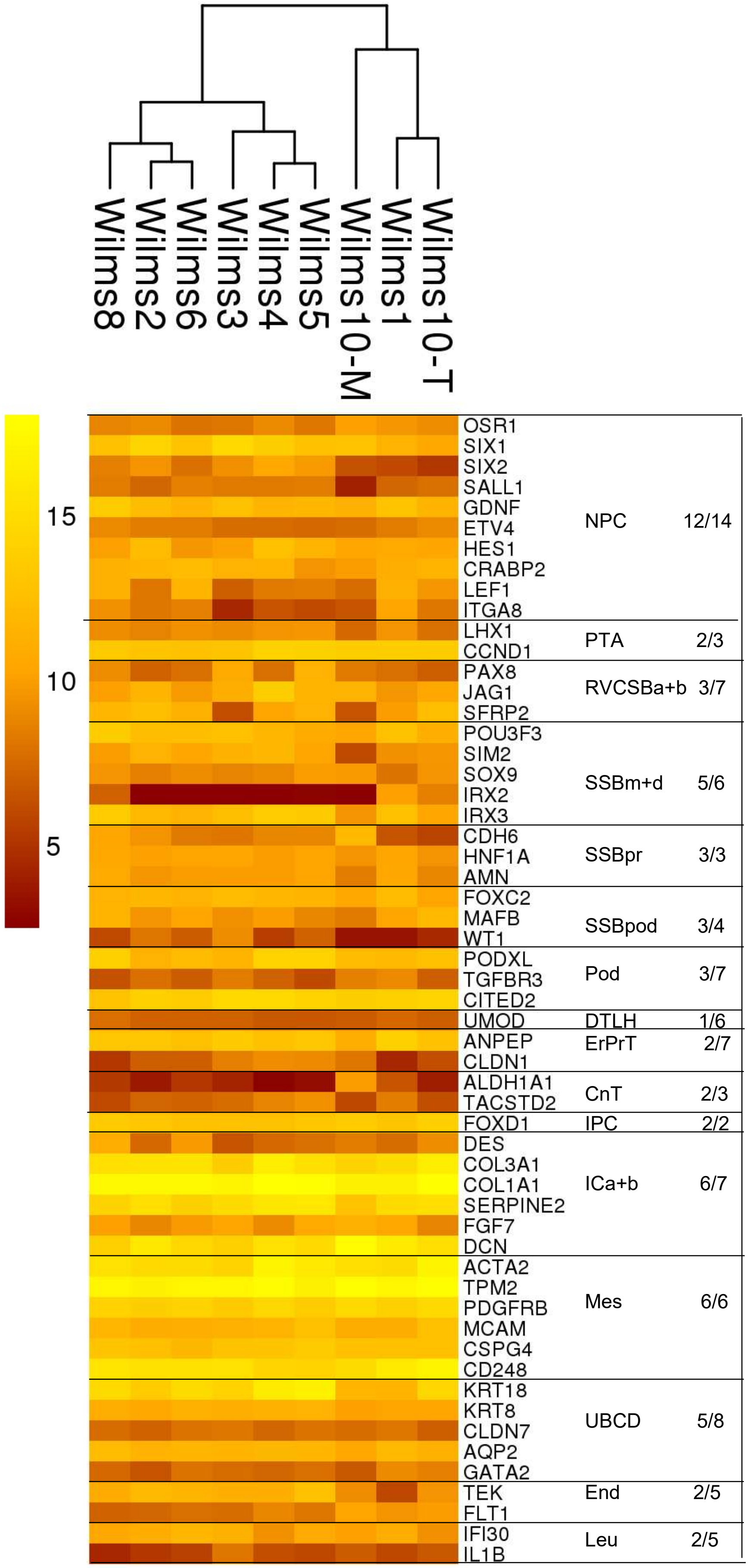

S5 Fig. Heat map of expression of literature genes [43] in the Wilms cell lines. One gene, *GDNF* is present in two clusters, NPC and IPC, but only listed in NPC.

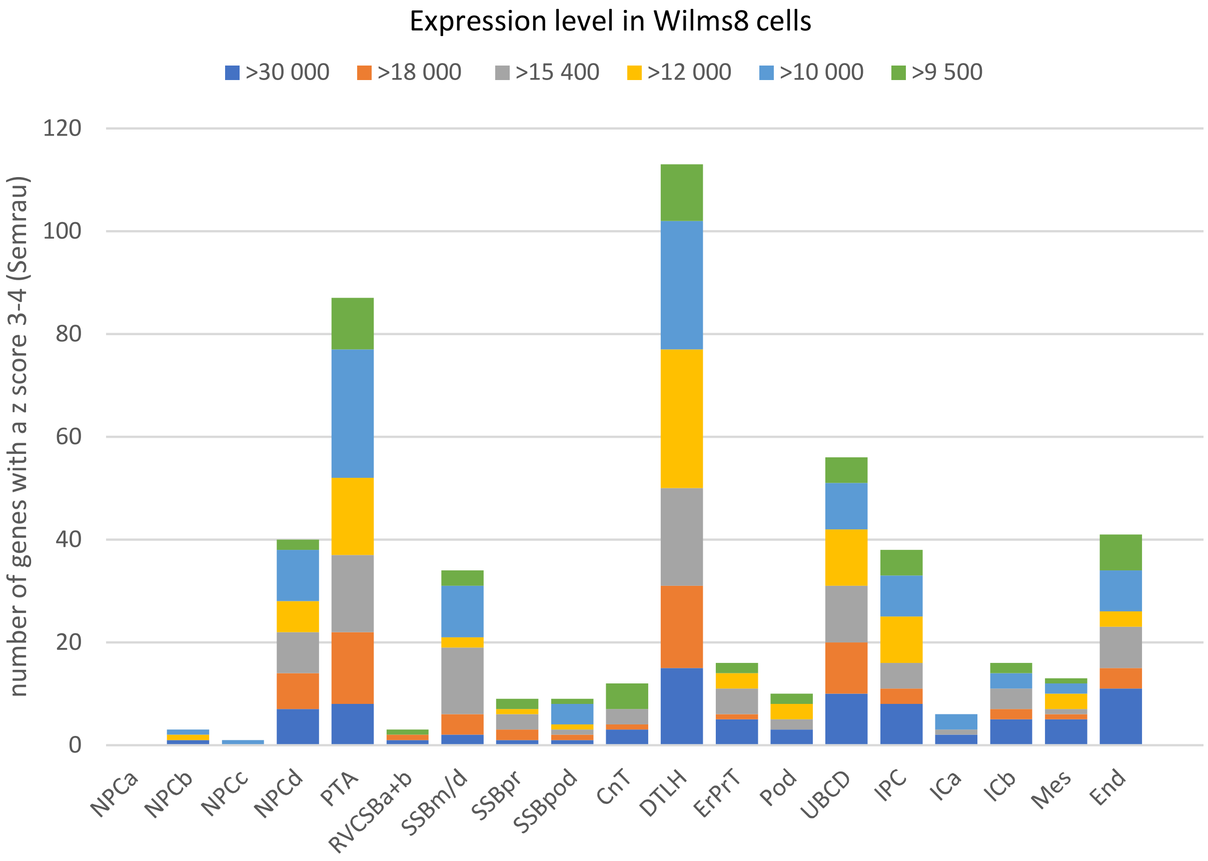

S6 Fig. Highest expressed genes in the Wilms8 cell line and classification to compartments. Here the number of genes with a Z-score expression in normal human fetal kidney between 3 and 4 (Semrau Database) are shown that are highly expressed in Wilms8. The different colors indicate the expression level.

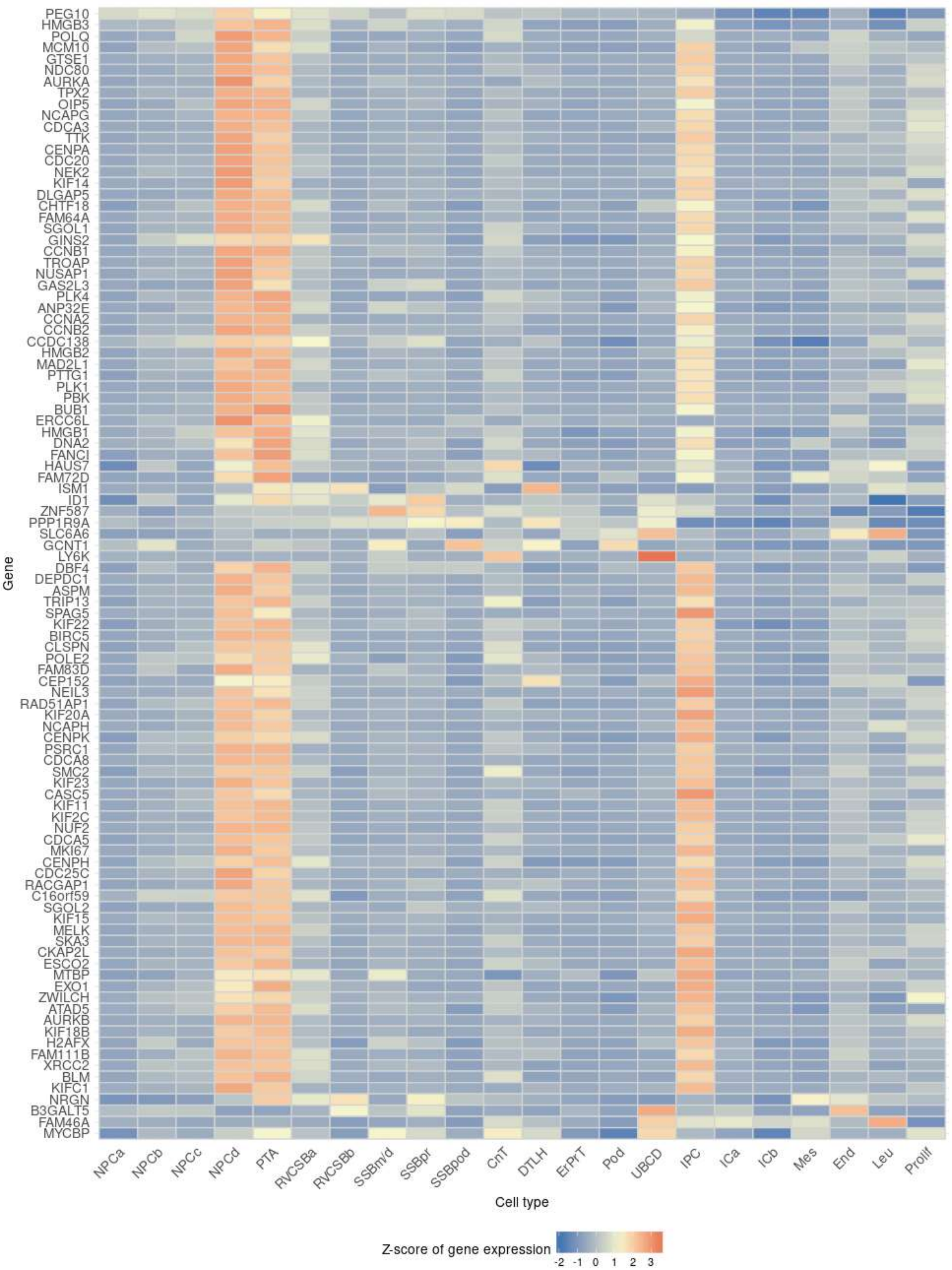

S7 Fig. Genes expressed higher in group2 cell lines and their classification to normal kidney compartments. Here the genes with a p-value <0.05, expression >200 and a ratio of >1.33 were analyzed for their allocation to normal human fetal kidney compartments. The result shows the expression of these genes in normal kidney, not in the Wilms cell lines (Semrau database).

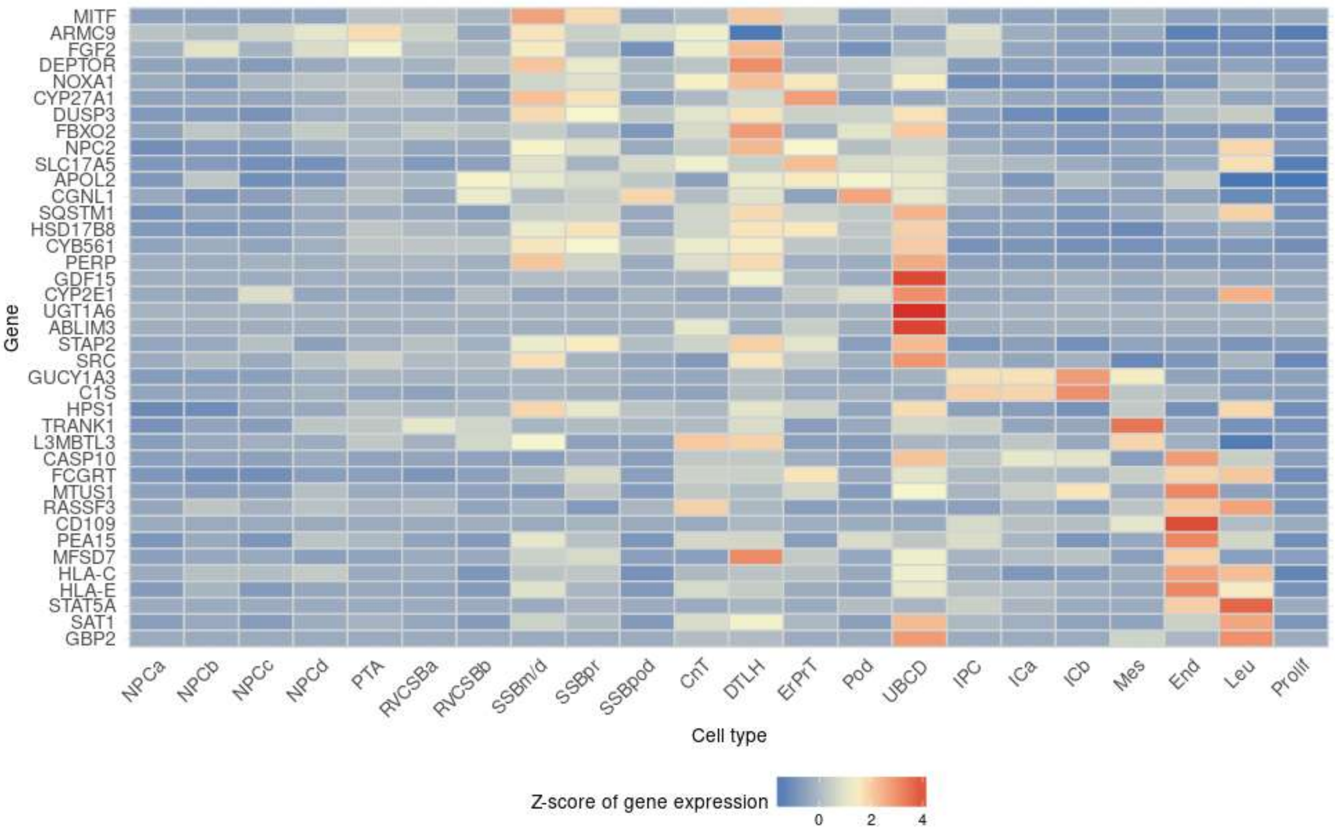

S8 Fig. Genes expressed lower in group 2 cell lines and their classification to normal kidney compartments. The genes with a significant lower expression in group 2 ($p<0.05$, expression >200 and a ratio of <0,75) were studied for their expression in normal human fetal kidney (Semrau database).

A

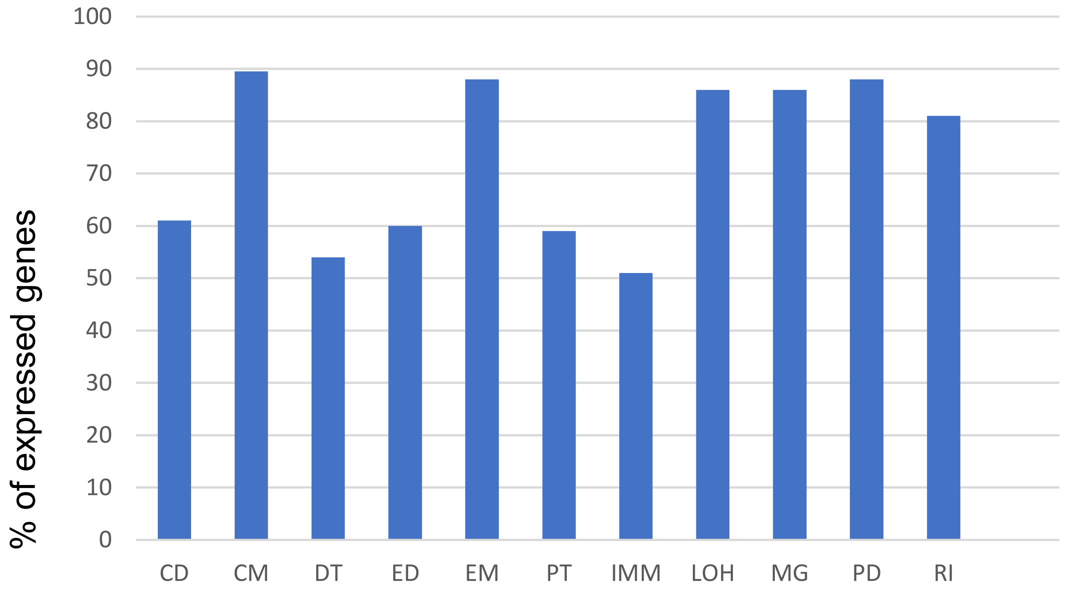

B

cluster	Number of genes	Expressed in Wilms >1000
CD collecting ducts	197	112 (61%)
CM cap mesenchyme	471	422 (89,5%
DT distal convoluted tubule	68	37 (54%)
ED endothelial cells	166	100 (60%)
EM extraglomerular mesangium	43	38 (88%)
PT proximal tubules	519	305 (59%)
IMM immune cells	245	127 (51%)
LOH loop of henle	93	80 (86%)
MG mesangium	101	86 (86%)
PD podocytes	43	38 (88%)
RI renal interstitium	53	43 (81%)

S9 Fig. Expression of genes from the 11 Wang clusters [15] in Wilms cell lines. A) percentage of genes from each compartment expressed in Wilms cell lines, cut off >1000 in any of the cell lines. **B)** The numbers of genes in each compartment and percentages of expressed genes in Wilms cell lines.
