# Supplementary material for: Gene expression studies of WT1 mutant Wilms tumor cell lines in the frame work of published kidney development data reveals their early kidney stem cell origin
Source: PLoS One. 2023 Jan 23;18(1):e0270380. doi: 10.1371/journal.pone.0270380 (PMC9870146; doi:10.1371/journal.pone.0270380)
Supplement: S1 Table — (DOCX) [file pone.0270380.s002.docx]

**S1 Table. Key genes for each Lindström cluster and highest expressed genes in Wilms cell lines in these clusters**

| cluster | Number of genes | Key genes for each cluster [32].  Green: not expressed in Wilms | Expressed in Wilms >1000 | Genes with highest expression in cluster  For full results see Table S2 |
| --- | --- | --- | --- | --- |
| 1 Immune response | 846 | *HLA-DRA, CCL3, SRGN* | 482 (56.9%) | n.a. |
| 2 ECM | 797 | *LUM, SFRP2, DCN* | 634 (79.3%) | *LGALS1, ANXA2, VIM, CD63, PTRF, S100A6, COL6A1* |
| 3 dev vasculature | 933 | *PLVAP, GNG11, TIE1* | 678 (88.3%) | *LGALS1, ANXA2, PTRF, S100A6, ITGB1, TUBB6* |
| 4 NP | 1149 | *LYPD1, DAPL1, SIX1, CITED1* | 903 (78.6%) | *BCAM, UCHL1, MIF, XRCC6, HINT1, VHL, HN1* |
| 5 Differentiating NP | 1030 | *MAL, ALDH1A1, KRT18, GATA3* | 689 (66.9%) | *BCAM, UCHL1, MIF, XRCC6, IER2, HINT1, HN1* |
| 6 Differentiating NP | 966 | *CDH6, EMX2, LRP2* | 551 (57%) | *BCAM, HSP90B1, GSTP1, GPX1, TUBB4B, IGFBP7* |
| 7 cell cycle | 890 | *HIST1H4C, HIST1A1A* | 721 (80.9%) | *TUBA1B, HMGN2, TUBA1C, TUBB6, TPM2, HS2ST1* |
| 8 cell cycling | 860 | *CENPF, TOP2A* | 707 (82.2%) | *LGALS1, CYB5R3, TUBA1B, SEPT9, HMGN2, TUBA1C* |
| 9 Interstitial | 300 | *REN, MGP, GATA3* | 194 (64.7%) | *CD63, ITGB1, TPM2, LMNA, COL4A2, TPM1* |
| 10 IP | 185 | *MEIS1, TAGLN, POSTN* | 140 (75.7%) | *PTRF, COL6A1, TPM2, HOXA3, CYR61, NGFRAP1* |
| 11 IP | 284 | *SULTE1, TCF21, ALDH1A2* | 216 (75.8%) | *LGALS1, VIM, TPM2, TGFBI, CYR61, COL4A2* |
| 12 IP | 308 | *VCAM1, FABP2, ANGPT1* | 244 (79.2%) | *LGALS1, CD63, COL6A1, ITGB1, COL1A1, SPARC* |
| Cluster4a | 222 | *CITED1, EYA1, ITGA8, TMEM100, MEOX1, ROBO2* | 190 (85.6)>200 | \| *COL6A1, COL1A2, NGFRAP1, MYL12A, GRN, VHL, SSR2, TPM1* \| \| --- \| |
| Cluster4 b | 59 | *ID1, MEG3, ARID5B, DAPL1* | 45 (76.3%)>200 | *VIM, B2M, SH3PXD2, CHMP2A, ID1, SERPINH1, PLK2, MEG3* |
| Cluster 4c | 271 | *LHX1, PAX8, TCF21, CCND1, PODXL* | 226 (83.4%) | *ANXA2, CD63, BCAM, SPARC, HSP90B1, LMNA, MXRA8, ATP5E* |
| Cluster 4d | 1380 | *HMGB2, CENPF, TOP2A, CCND1, UBE2C, NUSAP1* | 1246 (90.3%) >200 | *OAZ1, TUBA1B, HMGN2, TUBA1C,*  *GPX1, CHCHD2, MYL12A, COL4A2* |
