# Supplementary material for: Gene expression studies of WT1 mutant Wilms tumor cell lines in the frame work of published kidney development data reveals their early kidney stem cell origin
Source: PLoS One. 2023 Jan 23;18(1):e0270380. doi: 10.1371/journal.pone.0270380 (PMC9870146; doi:10.1371/journal.pone.0270380)
Supplement: S4 Table — (DOCX) [file pone.0270380.s005.docx]

**S4 Table: Marker genes for each Hochane cluster and highest expressed of genes in Wilms cell lines**

| cluster | Number of genes in cluster | TOP 4 genes from marker gene set  Green: not expressed | Expressed in Wilms >1000 | Genes with highest expression in Wilms cells in cluster, for complete list of expressed genes in clusters see Table S3 |
| --- | --- | --- | --- | --- |
| *NPCa* | *69* | *RSPO3, NKAIN3, FAM213A, TMEM100* | 38 (55.9%) | *UCHL1, TUBA1A, PCBP4, UBE2E3, HOXB2* |
| *NPCb* | *30* | *CACYBP, MRPL18, ZFAND2A*  *DNAJB1* | 22 (75.9%) | *HSPA8, IER2, HSP90AA1, HSPA1A, DNAJA1* |
| *NPCc* | *4* | *CRABP2, HAS2, MDK, HOXC6* | 3 (75%) | *CRABP2, HAS2, MDK* |
| *NPCd* | *85* | *CENPF, HMGB2, NUSAP1, CCNB2* | 80 (95,2%) | *HN1, STMN1, H2AFZ, SRSF3, HIST1H4C, H2AFZ* |
| *UBCD* | *218* | *ADH1C, PHLDA2, ELF5, CAPS* | 117 (53.9%) | *S100A6, RPL41, GSTP1, TUBA4A, UQCRQ* |
| *SSBpr* | *33* | *C1orf210, PIFO, CFAP126, C14orf105* | 16 (48.5%) | *ID1, CEBPD, CETN2, PCSK1N, TGIF1* |
| *SSBpod* | *156* | *OLFM3, IL1R1, PPIB, C17orf58* | 120 (76.9%) | *XRCC6, TPM1, SLC25A3, SEC13, EIF4A1, BTF3* |
| *SSBm/d* | *32* | *MITF, IRX1, CD24, TSPAN15* | 23 (71.9%) | *EEF1A1, GLTSCR2, SLC25A25, AKIRIN1, MITF* |
| *RVCSBa* | *32 (a+b) combined* | *SFRP2, CCND1, LHX1, LAMP5* | 18 (56.3%) (a+b) | *HNRNPA1, GLIPR2, CCND1, BEX1, APEX1, FBXO17, CCND1 (a+b)* |
| *RVCSBb* |  | *PAX8, KLK6, EMID1, PCP4* |  |  |
| *PTA* | *44* | *H2AFY, CALCA, RAN, RANBP1* | 36 (81.8%) | *PPIA, RAN, YBX1, FBL, CSRP2, SSRP1, HNRNPD, ACAT2* |
| *prolif* | *33* | *RPL22, RPS3A, RPL35, SNRPD2* | 33 (100%) | *RPL35, MIF, GAPDH, NDUFS5, RPS3A, ENO1, SNRPD2* |
| *Mes* | *243* | *TINAGL1, REN, NDUFA4L2, GNAS* | 166 (68.3%) | *TPM2, MT2A, CD99, GNAS, ATP5E, EIF3D, CD248* |
| *Leu* | *245* | *CD53, LST1, LSP1, CORO1A* | 126 (51.4%) | *FTH1, GPX1, BRI3, UBA52, S100A4* |
| *CnT* | *50* | *AKR1B1, MUC6, STXBP6, KRT17* | 24 (48%) | *SLIT2, HMGA1, AKR1B1, STXBP6, BCAT1, TWIST1, CLIP, CITED4* |
| *DTLH* | *260* | *MAL, TFCP2L1, HOXD8, SLC12A1* | 156 (60%) | *COL6A1, LDHB, ATP5G3, ATP5L, ALDOA, GPI, ATP5O, ATP5H* |
| *End* | *322* | *ECSCR, EGFL7, RAMP2, ICAM2* | 205 (63.7%) | *PTRF, EEF1D, MYL12A, HLA-C, THY1, GUK1* |
| *ErPrT* | *303* | *GLYAT, CUBN, SMIM24, APOE* | 150 (49.5%) | *TUBB4B, FTL, TPT1, DBI, CLTC, RP1, TXN* |
| *IPC* | *162* | *RPS8, COL1A2, CDC42EP5, COL1A1* | 142 (87.7%) |  |
| *ICa* | *6* | *RPS12, TAGLN, FGF7, NUPR1* | 6 (100%) | *RPS12, TAGLN, FGF7, NUPR1* |
| *ICb* | *190* | *SULT1E1, MOXD1, SFRP1, PRSS23* | 141 (74.2%) | *COL1A2, CDC42EP5, COL1A1, SSR2, NUPR1, FSTL1, DCN, TAGLN, COL6A3, LAPTM4A, APCDD1* |
| *Pod* | *1047* | *THSD7A, PTPRO, MPP5, TPPP3* | 888 (85%) | *OAZ1, CD63, DRD4, VIM, ANXA2, UBC, BCAM, ACTB* |
