# Supplementary material for: Gene expression studies of WT1 mutant Wilms tumor cell lines in the frame work of published kidney development data reveals their early kidney stem cell origin
Source: PLoS One. 2023 Jan 23;18(1):e0270380. doi: 10.1371/journal.pone.0270380 (PMC9870146; doi:10.1371/journal.pone.0270380)
Supplement: S6 Table — (DOCX) [file pone.0270380.s007.docx]

**S6 Table. Marker genes from Wang clusters and highest expressed genes in Wilms cell lines**

| cluster | Number of genes | Marker genes for cluster  Green: not expressed in Wilms cells | Expressed in Wilms >1000 | 8 highest expressed genes in Wilms cells from Wang cluster >1000, same colours indicate if present in different clusters, for full list see Table S5 |
| --- | --- | --- | --- | --- |
| CD collecting ducts | 197 | *AQP2, AQP3* | 112 (61%) | *ANXA2, S100A6, GRN, DSTN, TMBIM6, RTN3, PGK1, S100A10,* |
| CM cap mesenchyme | 471 | *SIX2 , NNAT, EXA1, COL2A1, MEOX1, PAX2 CITED1* | 422 (89,5% | *UCHL1, TUBB4B, XRCC6, RAB34, CCT4, HINT1, HN1, PSBM1* |
| DT distal convoluted tubule | 68 | *CLCNKB, POU3F3* | 37 (54%) | *S100A13, TNRFSF12A, ATPIF1, MRPS6, LRPAP1, KRT18, KRT19, OCIAD2* |
| ED endothelial cells | 166 | *CDH5, KDR, GPX3, APOE, BHMT* | 100 (60%) | *ANXA2, S100A6, ITGB1, FN1, COTL1, RHOC, CAV1, VAT1* |
| EM extraglomerular mesangium | 43 | *CXCL12, PDGFRB, REN* | 38 (88%) | *COL1A1, TPM2, TPM1, FSTL1, CALD1, COL6A3, CD248, COL5A2* |
| PT proximal tubules | 519 | *LRP2, CUBIN, BHMT* | 305 (59%) | *BCALM, NFE2L1, TUBB4B, HINT1, ATP6V0C, ATP5G3, DBI, HERPUD1* |
| ER erythrocytes | 67 | *HBQ1, HEGMN* |  | *n.a.* |
| ER-C contaminated cells | n.a. |  |  | *n.a.* |
| IMM immune cells | 245 | *CD53, SRIN, HSPE1* | 127 (51%) | *GPX1, COTL1, GRN, ARPC2, ANXA1, ARPC3, CTSB, NPC2* |
| LOH loop of henle | 93 | *UMOD, POU3F3, HSPA1B, HSPA1A, HSPE1* | 80 (86%) | *LMNA, CYR61, ATF4, IER2, IGFBP7,*  *HSPD1, HERPUD1, THBS1* |
| MG mesangium | 101 | *PDGFRB, FSTL1, LHFP, LOX, LAMA4* | 86 (86%) | *ANXA2, PTRF, S100A6, FSTL1, FN1, DKK3, TAGLN, CALD1,* |
| PD podocytes | 43 | *NPHS1, NPHS2, DCDC2* | 38 (88%) | *UCHL1, HINT1, ATP5G3, CUTA, S100A13, CBS, RTN3, UQCRH* |
| RI renal interstitium | 53 | *SFRP1, MEIS1, PDGFRA, MOXD1, PTN* | 43 (81%) | *TPM2, TGFBI, TPM1, FSTL1, IGFBP7, CALD1, COL6A3, DCN* |
